# Draft genome sequences of four *Morganella morganii* strains isolated from Colombian colorectal cancer patient stool specimens

**DOI:** 10.1128/mra.01077-23

**Published:** 2024-01-24

**Authors:** Carolina Hernández-Castro, Alejandro Múnera Duque, Juan Pablo Hoyos Burgos, Miguel Ángel Toro Londoño, Sonia del Pilar Agudelo López, David Carmena, Sergio Sánchez

**Affiliations:** 1 Parasitology Group, Faculty of Medicine, Academic Corporation for the Study of Tropical Pathologies, University of Antioquia, Medellín, Colombia; 2 Reference and Research Laboratory on Parasitology, Spanish National Centre for Microbiology, Health Institute Carlos III, Madrid, Spain; 3 Department of Surgery, Faculty of Medicine, University of Antioquia, Medellín, Colombia; 4 Hospital Alma Máter de Antioquia, Medellín, Colombia; 5 CIBERINFEC, ISCIII - CIBER Infectious Diseases, Health Institute Carlos III, Madrid, Spain; 6 Reference and Research Laboratory on Food and Waterborne Bacterial Infections, Spanish National Centre for Microbiology, Health Institute Carlos III, Madrid, Spain; University of Maryland School of Medicine, USA

**Keywords:** *Morganella morganii*, colorectal cancer

## Abstract

We report the draft genome sequences of four *Morganella morganii* strains isolated from the stools of four patients diagnosed with colorectal cancer (CRC) in Medellín, Colombia. These genomes represent an important addition to the limited number of genomes of *M. morganii* strains originating from CRC patients currently available.

## ANNOUNCEMENT

*Morganella morganii* is a Gram-negative, facultative anaerobic commensal bacterium of the human intestinal tract recognized as an important opportunistic pathogen (1) in a wide range of nosocomial and community-acquired infections (2–4). In addition, it has been shown that *M. morganii* is enriched in the gut microbiota of inflammatory bowel disease and colorectal cancer (CRC) patients and it has even been shown to produce a previously undescribed family of genotoxins, termed the indolimines, and causing increased intestinal permeability and exacerbated colon tumorigenesis in gnotobiotic mice (5). However, to the best of our knowledge, only one *M. morganii* strain isolated from CRC patient stool specimens has been sequenced so far (6); hence, additional genomes are needed to facilitate comparative genomic analyses and to elucidate the potential role of *M. morganii* in the development of CRC.

Strains were isolated in 2022 from the stools of four adult males diagnosed with CRC and hospitalized after colonoscopy in Medellín, Colombia. Real-time PCR based on the 16S rRNA gene was used to detect *M. morganii* from stool specimens (7). Positive specimens were plated onto MacConkey agar (Becton Dickinson, USA) and, following overnight incubation at 37°C, a maximum of ten lactose-negative (colorless) colonies per plate were randomly chosen and identified by biochemical methods (API 20E, BioMérieux, France). Definitive identification of the presumptive *M. morganii* colonies was based on 16S rRNA gene PCR and sequencing (8). According to NCBI BLASTN results, the best match accession numbers were MN807694 (strains IPS008 and IPS040), KM269029 (IPS012), and MN006020 (IPS030).

*Morganella morganii* strains from our laboratory stocks were overnight cultivated in tryptic soy agar (Becton Dickinson) at 37°C. Genomic DNA was extracted and purified using the NZY Tissue gDNA Isolation Kit (NZYTech, Portugal), according to the manufacturer’s instructions. A DNA library was generated using the Illumina DNA Prep Kit (Illumina, USA), according to the manufacturer’s instructions, and whole-genome sequencing (WGS) was performed with the Illumina NextSeq 500 platform (Illumina) using 300 cycles and generating 150 bp paired-end reads. The reads were trimmed and filtered according to quality criteria using FastP v0.23.2 and FastQC v0.11.9, respectively (9). The quality-filtered reads were assembled using Unicycler v0.4.8 (10) with *Morganella morganii* strain AR_0057 as the reference genome (accession number NZ_CP027177.1). Contigs shorter than 200 bp were removed to ensure sequencing quality and to meet the requirements for uploading to the NCBI Genome Database. All software was used with default settings unless otherwise specified. Complete genome sequences were obtained for all four *M. morganii* strains and genomes were annotated using the NCBI Prokaryotic Genome Annotation Pipeline v6.6. Detailed information about WGS parameters, assembly, and genome characteristics for individual strains is shown in Table 1.

**TABLE 1:** Whole-genome characteristics of the sequenced *Morganella morganii* strains^*a*^

	*Morganella morganii* strain			
	IPS008	IPS012	IPS030	IPS040
No. of reads	5,492,226	5,927,468	5,012,278	5,871,056
Depth of coverage	155×	170×	131×	160×
GC content (%)	51.00	51.14	51.14	50.93
Total length (bp)	3,830,508	3,848,985	3,910,626	3,912,298
Genome fraction (%)	81.06	86.07	85.66	81.31
Number of contigs	18	23	24	22
Contig N50	473,353	402,904	378,793	682,475
Contig L50	2	4	3	2
No. of predicted genes	3,669	3,705	3,744	3,795
No. of RNA genes	77	82	79	78
No. of pseudogenes	44	34	38	47
BioSample number	SAMN37692576	SAMN37692577	SAMN37692578	SAMN37692579

^*a*^ All statistics are based on contigs of size ≥ 500 bp.

Approval of the study protocol was obtained from the Ethics Committee of the Medical Research Institute [Faculty of Medicine, University of Antioquia (Ref. 00613.05.2021)], and the Technical Research Committee of the Hospital Alma Máter de Antioquia (Ref. IN32-2021).

## Data Availability

This whole-genome shotgun project has been deposited at DDBJ/ENA/GenBank as BioProject PRJNA1024498 under the accession numbers JAWLLN000000000 (strain IPS008), JAWLLO000000000 (strain IPS012), JAWLLP000000000 (strain IPS030), and JAWLLQ000000000 (strain IPS040). The versions described in this paper are JAWLLN010000000, JAWLLO010000000, JAWLLP010000000, and JAWLLQ010000000, respectively. Corresponding SRA data were deposited under accession numbers SRR26288169, SRR26288168, SRR26288167, and SRR26288166.

## References

[1] Liu H, Zhu J, Hu Q, Rao X. 2016. Morganella morganii, a non-negligent opportunistic pathogen. Int J Infect Dis 50:10–17. doi:10.1016/j.ijid.2016.07.006 27421818

[2] Learman BS, Brauer AL, Eaton KA, Armbruster CE. 2019. A rare opportunist, Morganella morganii, decreases severity of polymicrobial catheter-associated urinary tract infection. Infect Immun 88:e00691-19. doi:10.1128/IAI.00691-19 31611275 PMC6921659

[3] Lin TY, Chan MC, Yang YS, Lee Y, Yeh KM, Lin JC, Chang FY. 2015. Clinical manifestations and prognostic factors of Morganella morganii bacteremia. Eur J Clin Microbiol Infect Dis 34:231–236. doi:10.1007/s10096-014-2222-8 25107625

[4] Chang HY, Wang SM, Chiu NC, Chung HY, Wang HK. 2011. Neonatal Morganella morganii sepsis: a case report and review of the literature. Pediatr Int 53:121–123. doi:10.1111/j.1442-200X.2010.03241.x 21342342

[5] Cao Y, Oh J, Xue M, Huh WJ, Wang J, Gonzalez-Hernandez JA, Rice TA, Martin AL, Song D, Crawford JM, et al.. 2022. Commensal microbiota from patients with inflammatory bowel disease produce genotoxic metabolites. Science 378:eabm3233. doi:10.1126/science.abm3233 36302024 PMC9993714

[6] Stepanovica M, Zepeda-Rivera MA, McGlinchey AS, Baryiames AA, Jones DS, LaCourse KD, Bullman S, Johnston CD. 2022. Complete genome sequence of Morganella morganii CTX51T, isolated from a human cecal adenocarcinoma. Microbiol Resour Announc 11:e0006622. doi:10.1128/mra.00066-22 35254123 PMC9022561

[7] Fukumoto H, Sato Y, Hasegawa H, Saeki H, Katano H. 2015. Development of a new real-time PCR system for simultaneous detection of bacteria and fungi in pathological samples. Int J Clin Exp Pathol 8:15479–15488. 26823918 PMC4713704

[8] Marín M, Garcia-Lechuz JM, Alonso P, Villanueva M, Alcalá L, Gimeno M, Cercenado E, Sánchez-Somolinos M, Radice C, Bouza E. 2012. Role of universal 16S rRNA gene PCR and sequencing in diagnosis of prosthetic joint infection. J Clin Microbiol 50:583–589. doi:10.1128/JCM.00170-11 22170934 PMC3295163

[9] Chen S, Zhou Y, Chen Y, Gu J. 2018. fastp: an ultra-fast all-in-one FASTQ preprocessor. Bioinformatics 34:i884–i890. doi:10.1093/bioinformatics/bty560 30423086 PMC6129281

[10] Wick RR, Judd LM, Gorrie CL, Holt KE. 2017. Unicycler: resolving bacterial genome assemblies from short and long sequencing reads. PLoS Comput Biol 13:e1005595. doi:10.1371/journal.pcbi.1005595 28594827 PMC5481147

